# CircPTK2 (hsa_circ_0003221) Contributes to Laryngeal Squamous Cell Carcinoma by the miR-1278/YAP1 Axis

**DOI:** 10.1155/2021/2408384

**Published:** 2021-10-13

**Authors:** Zhendong Yang, Jianping Jin, Tao Chang

**Affiliations:** Department of E.N.T, The Ninth People's Hospital of Suzhou, Suzhou 215200, Jiangsu, China

## Abstract

Laryngeal cancer accounts for 20% of all head and neck malignancies. Laryngeal squamous cell carcinoma (LSCC) is the most common type of laryngeal cancer and is characterized by squamous differentiation, a high mortality rate, and poor prognosis. Accumulating studies have indicated that circular RNAs (circRNAs) are critical regulators in many cancers. CircPTK2 exerts an important regulatory role in several cancers. In this study, we aimed to elucidate the function of circPTK2 (hsa_circ_0003221) in LSCC. Through a series of investigations, we discovered that circPTK2 was significantly upregulated in LSCC tissues cells. Functionally, cell counting kit-8 (CCK-8) and flow cytometry analyses revealed that knockdown of circPTK2 suppressed LSCC cell viability and the cell cycle while promoting cell apoptosis. Notably, silencing circPTK2 inhibited tumor growth *in vivo*. Mechanistically, circPTK2 functioned as a molecular sponge of miR-1278 to upregulate YAP1 expression in LSCC cells. Moreover, YAP1 knockdown inhibited malignant phenotypes of LSCC cells. The rescue experiments showed that YAP1 overexpression reversed the effects of circPTK2 on LSCC cells. Therefore, we concluded that circPTK2 facilitates LSCC progression through the miR-1278/YAP1 axis.

## 1. Introduction

Laryngeal squamous cell carcinoma (LSCC), with high mortality and incidence rates, is one of the most prevalent subtypes of laryngeal carcinoma [1, 2]. The onset of LSCC is acute, and 60% of LSCC patients are diagnosed in an advanced stage [3]. The incidence of LSCC in China is approximately four times higher than that in the United States, with over 15,000 deaths each year [4]. Currently, surgery together with radiation and drug therapy is the main treatment strategy for LSCC. However, the survival rate of LSCC patients remains low due to high recurrence and distant metastasis [5, 6]. Therefore, new therapeutic methods for LSCC are needed. Targeted molecular therapy has shown a good promising effect in improving the prognosis of advanced patients [7]. Hence, it is important to reveal the potential molecular biomarkers for LSCC treatment.

Circular RNAs (circRNAs) are endogenous noncoding transcripts generated by pre-mRNA back splicing and are characterized by a covalently closed loop without 5′ caps and 3′ tails [8, 9]. Most circRNAs have no protein-coding potential, and only a small portion can be translated into polypeptides [10, 11]. Owing to their special structures, circRNAs are more stable than their linear mRNAs, which ensures their high conservation and abundance [12]. CircRNAs have been reported to be involved in the progression of human cancers at the transcriptional, posttranscriptional, and translational levels [13]. Moreover, circRNAs could act as competitive endogenous RNAs (ceRNAs) for miRNA sponges to affect the biological activity of their target mRNAs [14]. For example, circ0003998 increases cell proliferation and invasion in gastric cancer by targeting the miR-1205/E2F1 axis [15]. circ5615 facilitates colorectal cancer progression through upregulating TNKS by acting as a sponge of miR-149-5p [16]. An increasing number of studies have revealed the roles of circRNAs in LSCC. For example, circCORO1C promotes LSCC cell proliferation, migration, and invasion by regulating the let-7c-5p/PBX3 axis [17]. circ_0067934 predicts a poor prognosis of LSCC and promotes LSCC progression by sponging miR-1324 [18]. Silencing circFLNA suppresses LSCC cell migration by regulating miR-486-3p [19]. In this work, we investigated the molecular mechanisms of circPTK2 (hsa_circ_0003221) in LSCC with circBase (http://www.circbase.org). hsa_circ_0003221 with a spliced sequence length of 625 nt is a product of five back-spliced exons (exons 3, 4, 5, 6, and 7). CircPTK2 (hsa_circ_0005273) exerts oncogenic functions in colorectal cancer cell growth and may serve as a potential therapeutic target [20]. CircPTK2 (hsa_circ_0008305) inhibits the epithelial-mesenchymal transition process in non-small-cell lung cancer [21]. Additionally, circPTK2 (hsa_circ_0003221) facilitates bladder cancer cell proliferation and migration [22]. However, the biological role of circPTK2 in LSCC remains unknown.

This study focused on the role of circPTK2 and the regulatory mechanisms in LSCC progression. Our findings may provide a deeper understanding of the tumorigenic mechanism and suggest a potential target for LSCC treatment.

## 2. Materials and Methods

### 2.1. LSCC Tissue Samples

A total of 41 pairs of LSCC tissues and matched tissues (taken 1–3 cm from the edge of cancer tissues) were obtained from patients undergoing surgery at the Department of E.N.T, The Ninth People's Hospital of Suzhou. None of the patients received chemotherapy or radiotherapy before surgery. The tissue samples were diagnosed independently by two experienced clinical pathologists. Fresh specimens were immediately frozen in liquid nitrogen. This study was approved by the Institutional Ethical Review Committee of The Ninth People's Hospital of Suzhou, and each patient provided written informed consent.

### 2.2. Cell Culture

Human LSCC cell lines (SNU899 and SNU46) were commercially supplied by the Korean Cell Line Bank. The human bronchial epithelioid cell line 16HBE was purchased from Millipore (Boston, MA, USA). Cell lines were cultured in RPMI-1640 (Gibco, Grand Island, NY, USA) with 10% fetal bovine serum (FBS, Invitrogen, Carlsbad, CA, USA), 100 units/mL penicillin, and 100 *µ*g/mL streptomycin in a humidified incubator with 5% CO_2_ at 37°C.

### 2.3. Cell Transfection

Short hairpin RNAs (shRNAs) targeting circPTK2 (sh-circPTK2#1 or #2), YAP1 (sh-YAP1), and shRNA negative control (sh-NC) were supplied by GenePharma (Shanghai, China). The miR-1278 mimics and corresponding negative controls (NC mimics) were purchased from GenePharma. SNU46 and SNU899 cells were transfected with Lipofectamine 2000 (Invitrogen) following the manufacturer's protocol. For *in vivo* assays, lentiviral vectors containing sh-circPTK2 or the negative control were purchased from Hanbio Biotechnology (Shanghai, China) and then used to infect SNU46 cells.

### 2.4. Cell Counting Kit-8 (CCK-8) Assay

The assessment of LSCC cell viability was performed with a CCK-8 kit (Dojindo, Kyushu, Japan). All cells were incubated in 96-well plates. At 0, 24, 48, 72, and 96 h, 10 *µ*l CCK-8 solution was added to each well for 4 h of incubation. Subsequently, the optical density (OD) of cells was measured at 450 nm using a microplate reader.

### 2.5. Flow Cytometry Analysis

Two days after transfection, LSCC cells were trypsinized and washed with PBS. Next, the cells were resuspended in annexin-binding buffer, followed by staining with annexin V-FITC and propidium iodide (BD Biosciences, Hercules, NJ, USA) for 15 min in the dark. For cell cycle assessment, the collected cells were stained with annexin V-FITC. Finally, a FACScan flow cytometer (BD Biosciences) and FlowJo software (Tree Star, USA) were used for the corresponding detection.

### 2.6. Real-Time Quantitative PCR (RT-qPCR)

Total RNA was extracted using TRIzol reagent (Invitrogen). The RNA was reverse transcribed into complementary DNA (cDNA) using a Roche Reverse Transcription Kit (Shanghai, China) or TaqMan™ Advanced miRNA cDNA Synthesis Kit (Thermo Fisher, Waltham, MA, USA). A Thermal Cycler Dice Real-Time PCR System (TaKaRa, Dalian, China) was used for RT-qPCR. Using the 2^−ΔΔCt^ method, the expression of genes was analyzed. GAPDH or U6 acted as an internal control.

### 2.7. Agarose Gel Electrophoresis

Two percent agarose gel electrophoresis with TAE buffer was used to separate PCR products using a 100 bp DNA ladder (TransGen Biotech, Beijing, China). An Azure C600 imager (Azure Biosystems, Dublin, CA, USA) was used to observe the bands. For Sanger sequencing, PCR amplification products were excised from the agarose gel and purified using a GeneJET Gel Purification Kit (Thermo Fisher Scientific, USA). The nucleotide sequences of the purified fragments were determined by Sanger sequencing using standard approaches by Geneseed (Guangzhou, China) [23].

### 2.8. RNase R and Actinomycin D Treatment Assay

According to the protocols previously described [24], RNA was incubated with RNase (3 units per *μ*g, Geneseed Biotech, Guangzhou, China) for 30 min and LSCC cells were exposed to 2 *μ*g/mL Actinomycin D (HY-17559, MedChemExpress, Monmouth Junction, NJ, USA) for 0.5, 1, 2, 4, or 8 h. Then, the expression of circPTK2 and PTK2 was detected by RT-qPCR.

### 2.9. Western Blot Analysis

Proteins were obtained using RIPA lysis buffer (Beyotime, Shanghai). Afterwards, 10% sodium dodecyl sulfate-polyacrylamide gel electrophoresis was used to separate the proteins. The protein was then transferred onto polyvinylidene fluoride membranes (Millipore). After blocking with 5% skim milk, the membranes were incubated with primary antibodies at 4 °C overnight. Primary antibodies against cyclin A1 (ab53699), cyclin B1 (ab251892), cyclin D1 (ab40754), Bcl-2 (ab141523), Bax (ab32503), TP53 (ab202026), and GAPDH (ab181602) were purchased from Abcam Company (UK). Next, the membranes were incubated with the appropriate secondary antibodies for 2 h at room temperature. Pharmacia enhanced chemiluminescence was used to visualize the protein bands. GAPDH functioned as a loading control.

### 2.10. RNA Immunoprecipitation (RIP) Assay

RIP assays were performed using an RNA-Binding Protein Immunoprecipitation Kit (Millipore). Cell lysates in RIPA buffer were incubated with magnetic pellets covered with anti-Ago2, and anti-IgG was used as a negative control. Next, the immunoprecipitated RNA was purified by proteinase K. The purified RNA was analyzed by RT-qPCR.

### 2.11. Bioinformatics Analysis

The binding site between circPTK2 and miR-1278 was predicted with the website https://circinteractome.irp.nia.nih.gov/mirna_target_sites.html. The binding site between YAP1 and miR-1278 was predicted with the website http://starbase.sysu.edu.cn/.

### 2.12. Luciferase Reporter Assay

Wild-type (WT) circPTK2 and mutant circPTK2 sequences, as well as WT and MUT sequences from the 3′-untranslated regions (3′-UTR) of YY1, were synthesized and inserted into pGL3-basic reporter vector (Promega, Madison, WI, USA). LSCC cells were transfected with the reporter vectors together with miR-1278 mimics or NC mimics. After 48 h of transfection, the luciferase activity was measured by a dual-luciferase reporter assay system (Promega). The relative firefly luciferase activity was normalized to the Renilla luciferase activity, which served as an internal control.

### 2.13. Tumor Xenografts in Nude Mice

All animal experiments were conducted according to the Guide for the Care and Use of Laboratory Animals and were approved by the Ethics Committee of The Ninth People's Hospital of Suzhou. Twelve 6–8-week-old male BALB/*c* nude mice were purchased from Vital River Lab Animal Technology Company (Beijing, China). Approximately, 200 *μ*L of 5 × 10^6^ lenti-sh-circPTK2 (*n* = 6) or lenti-sh-NC SNU46 (*n* = 6) cells was subcutaneously injected into the right armpit of mice. The mice were anesthetized with an intraperitoneal injection of 75 mg/kg pentobarbital to minimize suffering. The tumor size was recorded every 4 days after injection, and the length (*L*) and width (W) of the tumors were measured. The tumor volume was calculated using the formula V = (*L*×*W*^2^) × 0.5. Twenty days after injection, the mice were euthanized with an intraperitoneal injection of 200 mg/kg pentobarbital and the tumors grown in nude mice of each group were resected and photographed.

### 2.14. Statistical Analysis

Data were analyzed using SPSS 20.0 Software (SPSS, Chicago, IL, USA). All data are expressed by the mean ± standard deviation. The differences in multiple groups were analyzed by one-way/two-way analysis of variance (ANOVA) followed by post hoc Dunnett's test (for comparisons with one control) and Tukey's test (for comparisons among various groups), and the differences between the 2 groups were analyzed by Student's *t*-test. All assays were carried out three times independently. A *p* value < 0.05 was considered statistically significant.

## 3. Results

### 3.1. Circular Characteristics of CircPTK2

**Figure 1:**
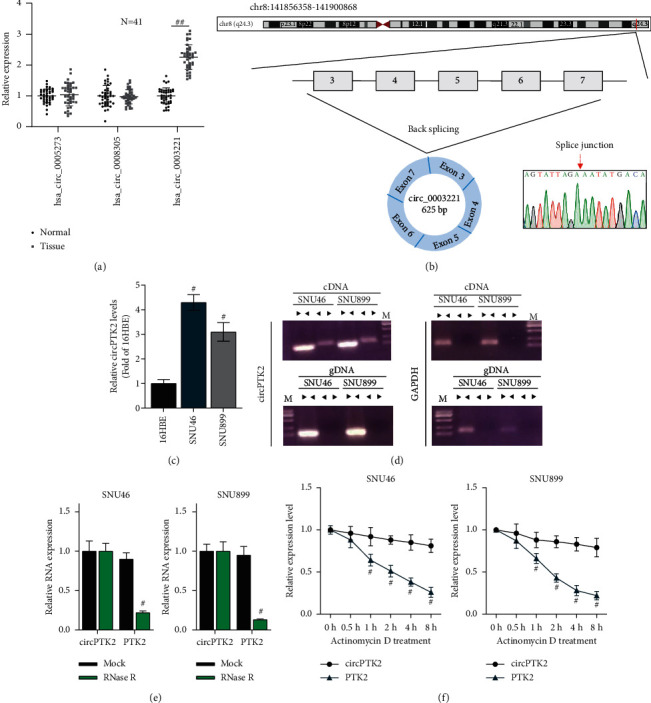
Circular characteristics of circPTK2. (a) The expression of hsa_circ_0005273, hsa_circ_0008305, and hsa_circ_0003221 in LSCC tissue samples was measured by RT-qPCR. (b) Schematic illustration of hsa_circ_0003221 formation and the results of Sanger sequencing. (c) CircPTK2 expression in SNU46 and SNU899 cells was measured by RT-qPCR. (d) Agarose gel electrophoresis revealed circular characteristics of circPTK2. cDNA and gDNA served as the templates. (e) The expression of circPTK2 and linear PTK2 after RNase R treatment. (f) RT-qPCR analysis of the abundance of circPTK2 and linear PTK2 in SNU46 and SNU899 cells treated with actinomycin D at the indicated times. ^#^*p* < 0.05; ^##^*p* < 0.05.

Three circRNAs (hsa_circ_0005273, hsa_circ_0008305, and hsa_circ_0003221) derived from the same pre-mRNA PTK2 were reported to participate in the progression of cancers, we aimed to test which circRNA is differentially expressed in LSCC samples. In this study, 41 pairs of LSCC tissues and adjacent normal tissues were used. As the results of RT-qPCR showed (Figure 1(a)), hsa_circ_0003221 expression was significantly upregulated in LSCC tissues compared with that in adjacent normal tissues, suggesting that hsa_circ_0003221 may play a role in LSCC progression. However, hsa_circ_0005273 and hsa_circ_0008305 had no significant change in the expression levels. Therefore, we chose hsa_circ_0003221 as the targeted circRNA to investigate its biological role in LSCC. According to the median expression of hsa_circ_0003221 in LSCC samples, the patients with LSCC were divided into a high-expression group (*n* = 21) and a low-expression group (*n* = 20). Fisher's exact test showed that a higher level of hsa_circ_0003221 was closely related to the clinical stage (*p* = 0.017) and lymph-node metastasis (*p* = 0.043). Its expression was not correlated with gender, age, smoking history, and *T* stage (*p* > 0.05 for all, Table 1). hsa_circ_0003221 is derived from exons 3, 4, 5, 6, and 7 regions within the PTK2 (protein tyrosine kinase 2) locus (human hg19 chr8:141856358–141900868), which is located on chromosome 8q24.3, and the spliced length is 625 bp. The head-to-tail splicing of exon 3 to exon 7 was confirmed by Sanger sequencing (Figure 1(b)). We further demonstrated that circPTK2 was more highly expressed in LSCC cells (SNU899 and SNU46) than in the human bronchial epithelioid cell line 16HBE (Figure 1(c)). CircRNAs are characterized by their stable structure due to the absence of a 5′ cap and a 3′ polyadenylated tail. Here, circPTK2 and PTK2 mRNA were amplified using divergent and convergent primers, respectively. Agarose gel electrophoresis results indicated that the divergent primers could amplify circPTK2 in only cDNA, while convergent primers amplified linear PTK2 in both cDNA and genomic DNA (gDNA) (Figure 1(d)). We then examined the stability of circPTK2 in LSCC cells. RNase R (a kind of exoribonuclease) treatment significantly reduced the expression of linear PTK2 but had no impact on the expression of circPTK2 (Figure 1(e)). Furthermore, we analyzed the half-life of linear PTK2 and circPTK2 using actinomycin D (an RNA transcription inhibitor) treatment, and the PCR analysis results showed that circPTK2 had a longer half-life than linear PTK2 in LSCC cells (Figure 1(f)). Overall, circPTK2 has a loop structure and is upregulated in OSCC.

### 3.2. CircPTK2 Knockdown Inhibits Cell Proliferation and Cell Cycle Progression in LSCC

**Figure 2:**
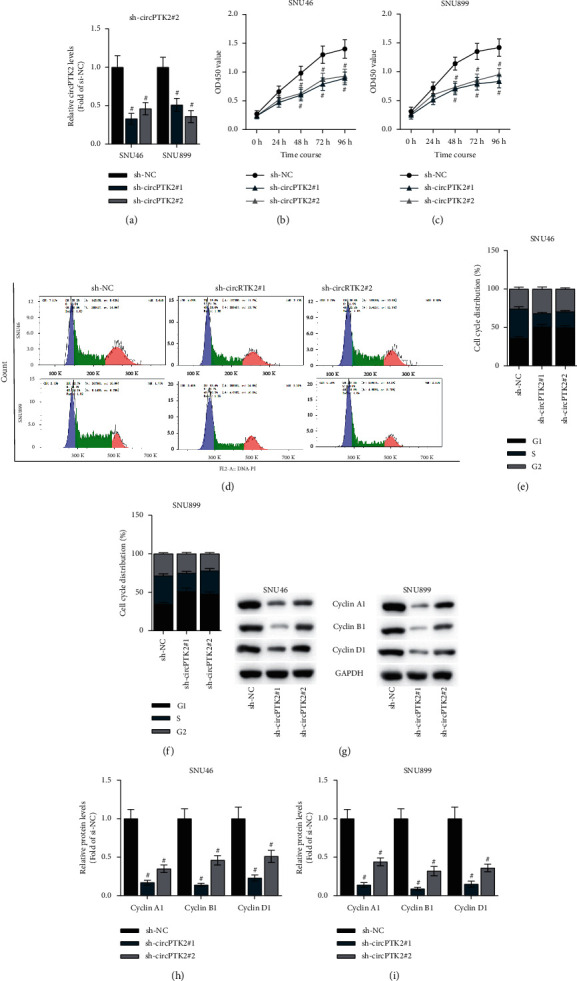
CircPTK2 knockdown inhibits cell proliferation and induces cell cycle arrest in LSCC cells. (a) RT-qPCR analysis was utilized to examine the knockdown efficiency of circPTK2 in SNU46 and SNU899 cells. (b, c) A CCK-8 assay was conducted to assess the viabilities of SNU46 and SNU899 cells. (d–f) Flow cytometry analysis was carried out to measure the cell cycle in LSCC cells. (g–i) The protein expression of Cyclin A1, Cyclin B1, and Cyclin D1 was measured by western blot analysis. ^#^*p* < 0.05.

Next, we investigated the biological function of circPTK2 in LSCC cells. First, circPTK2 was knocked down by sh-circPTK2#1 or sh-circPTK2#2 vector with sh-NC as a scramble control. As shown in Figure 2(a), the expression of circPTK2 in SNU46 and SNU899 cells was significantly reduced after transfection of sh-circPTK2#2, indicating that sh-circPTK2#1/2 could be used in subsequent assays. CCK-8 assay suggested that silencing circPTK2 suppressed the viabilities of SNU46 and SNU899 cells (Figures 2(b)–2(c)). Additionally, circPTK2 knockdown induced cell cycle arrest in the *G*0/*G*1 phases (Figures 2(d)–2(f)), suggesting that the cell cycle was inhibited by downregulated circPTK2 in LSCC cells. Subsequently, the levels of cell cycle-associated proteins (Cyclin A1, Cyclin B1, and Cyclin D1) in SNU46 and SNU899 cells were measured by western blot analysis, and the results showed the reduced levels of these proteins after circPTK2 knockdown (Figures 2(g)–2(i)). These findings demonstrated that silencing circPTK2 inhibits cell viability and cell cycle progression in LSCC.

### 3.3. CircPTK2 Knockdown Promotes LSCC Cell Apoptosis

**Figure 3:**
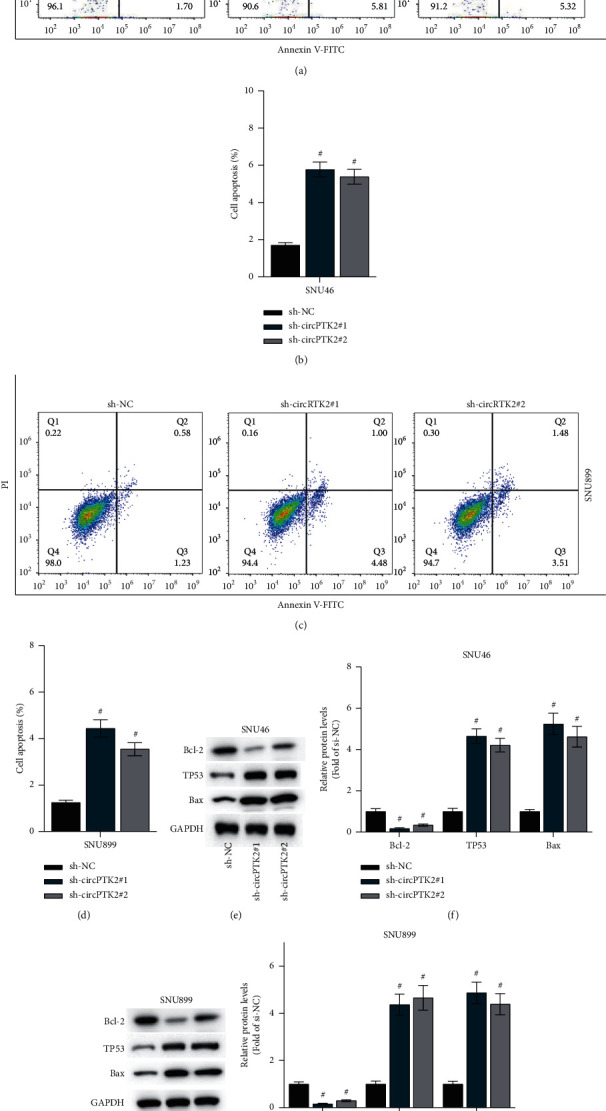
CircPTK2 knockdown promotes LSCC cell apoptosis. (a–d) The apoptosis of SNU46 and SNU899 cells was evaluated using flow cytometry analysis. (e–h) The protein levels of Bax, TP53, and Bcl-2 were tested by western blot analysis. ^#^*p* < 0.05.

We then investigated the effects of circPTK2 on LSCC cell apoptosis. From Figures 3(a)–3(d), inhibition of circPTK2 significantly increases the apoptosis rate of SNU46 and SNU899 cells. Furthermore, the levels of apoptosis-related proteins (Bax, TP53, and Bcl-2) were measured by western blot analysis, and the data suggested that downregulation of circPTK2 elevated the protein expression of Bax and TP53 but reduced that of Bcl-2 in SNU46 and SNU899 cells (Figures 3(e)–3(h)). Therefore, circPTK2 knockdown suppresses LSCC cell apoptosis.

### 3.4. CircPTK2 Downregulation Suppresses Tumor Growth *In Vivo*

**Figure 4:**
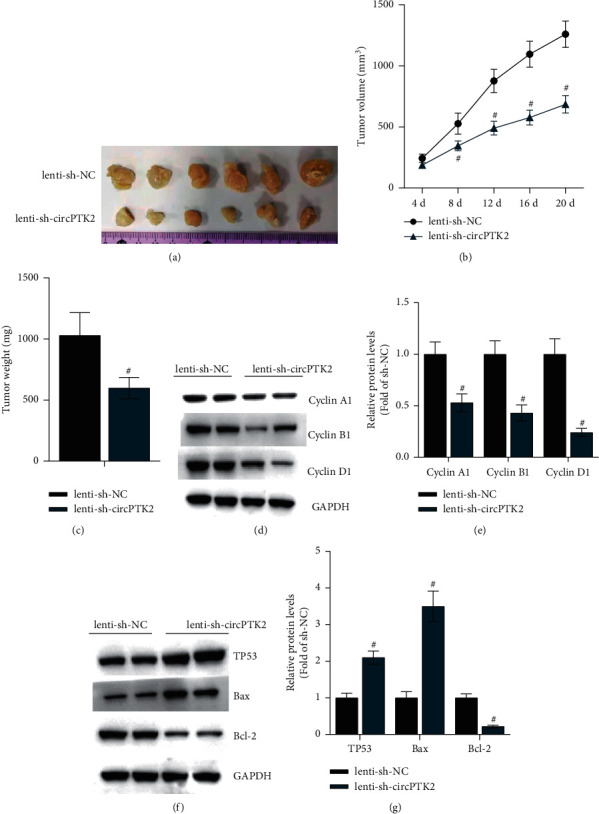
CircPTK2 knockdown suppresses xenograft growth in vivo. (a) The images of surgically removed tumors. (b, c) In the xenograft model, the volume and weight of tumors were recorded. (d–g) Western blot analysis was performed to analyze the protein expression of cell cycle-associated proteins (Cyclin A1, Cyclin B1, and Cyclin D1) and apoptosis-associated proteins (Bcl-2, Bax, and TP53) in the surgically removed tumors. ^#^*p* < 0.05.

Subsequently, we explored the function of circPTK2 *in vivo* by establishing a xenograft mouse model. As shown in Figure 4(a), circPTK2 depletion significantly decreased tumor growth. The volume and weight of tumors were significantly smaller in the lenti-sh-circPTK2 group than in the control group (Figures 4(b)–4(c)). The results of western blot analysis suggested that circPTK2 depletion suppressed the protein levels of Cyclin A1, Cyclin B1, and Cyclin D1 in the resected tumors (Figures 4(d)–4(e)). Additionally, the protein levels of Bax and TP53 were increased, while levels of Bcl-2 were decreased after circPTK2 suppression (Figures 4(f)–4(g)). In conclusion, knockdown of circPTK2 reduces the tumor growth of LSCC *in vivo*.

### 3.5. CircPTK2 Acts as a Sponge for miR-1278 to Upregulate YAP1 in LSCC Cells

**Figure 5:**
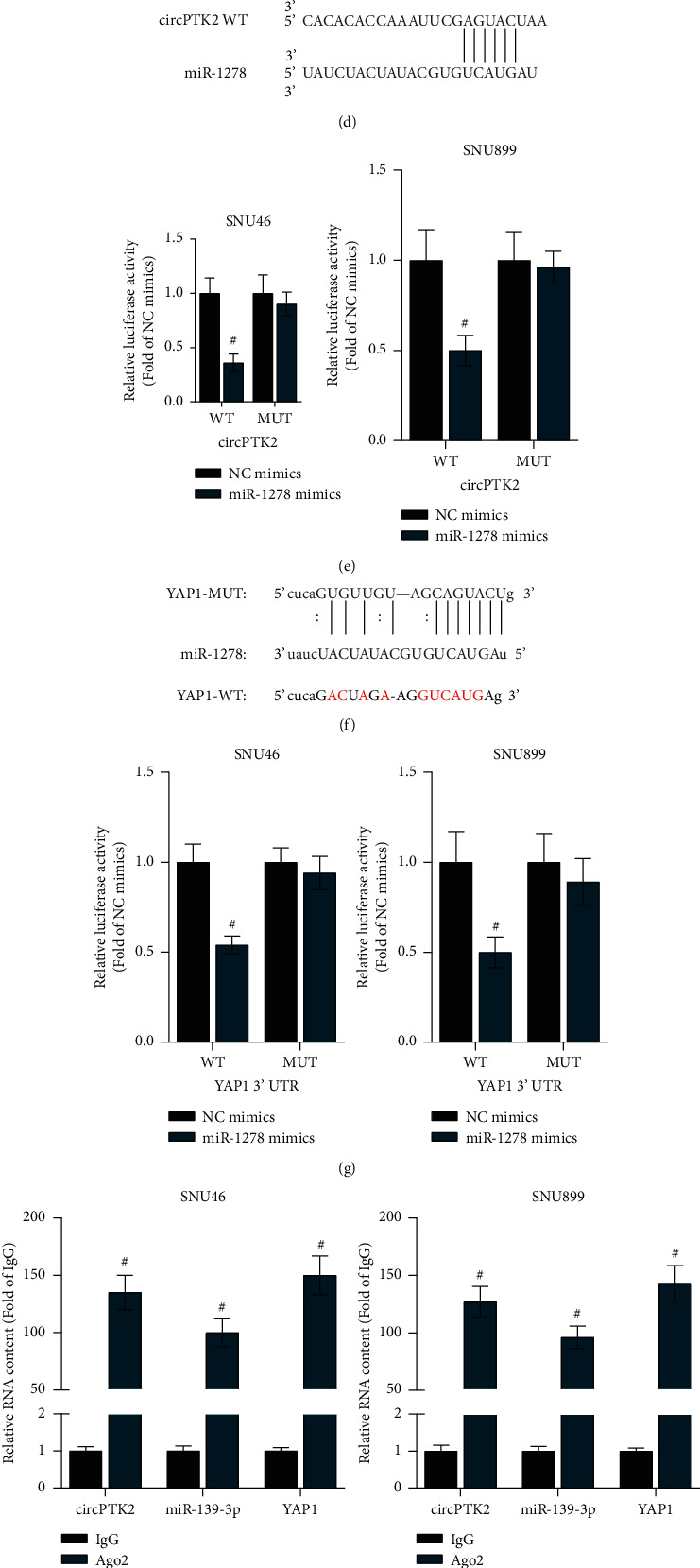
CircPTK2 binds to miR-1278 to upregulate YAP1 expression in LSCC cells. (a) RT-qPCR analysis of the expression of 5 candidate miRNAs in LSCC cells. (b) RT-qPCR analysis showed the influence of circPTK2 knockdown on miR-1278 expression. (c) RT-qPCR analysis tested the overexpression efficiency of miR-1278. (d) The predicted binding site between circPTK2 and miR-1278. (e) A luciferase reporter assay was conducted to identify the binding of circPTK2 and miR-1278. (f) The predicted binding site between YAP1 and miR-1278 was predicted from starBase. (g) A luciferase reporter assay was conducted to identify the binding of YAP1 to miR-1278. (h) RIP was performed to determine the enrichment of circPTK2, miR-1278, and YAP1 in Ago2 immunoprecipitation. (i, j) RT-qPCR and western blot analyses of YAP1 mRNA and protein levels in circPTK2-knockdown cells. (k) RT-qPCR analysis of YAP1 expression in cells transfected with the indicated plasmids.^#^*p* < 0.05.

A previous study pointed out that circPTK2 inhibits metastasis under the ceRNA pattern in non-small cell lung cancer [21], so we speculated that circPTK2 might act as a ceRNA in LSCC. Therefore, we explored whether circPTK2 could bind to specific miRNAs in LSCC. In light of the circular RNA Interactome [25], the top 5 miRNAs (miR-1278, miR-1279, miR-1322, miR-136, miR-139-3p) potentially binding to circPTK2 were identified. The RT-qPCR results indicated that only miR-1278 was downregulated in LSCC cells (Figure 5(a)). Additionally, the expression of miR-1278 was significantly upregulated after silencing circPTK2 (Figure 5(b)). Thus, miR-1278 was chosen for further assays. Next, we successfully overexpressed miR-1278 by transfection of miR-1278 mimics in SNU46 and SNU899 cells (Figure 5(c)). The binding site of miR-1278 on circPTK2 is shown in Figure 5(d). The luciferase activity of pmirGLO-circPTK2-WT was reduced by miR-1278 mimics. However, no marked change in the luciferase activity was found in the pmirGLO-circPTK2-MUT group, indicating that circPTK2 binds to miR-1278 at the predicted site (Figure 5(e)). By consulting the starBase database [26], YAP1 was predicted to be a potential target of miR-1278. The predicted binding sequence for miR-1278 binding to YAP1 is shown in Figure 5(f). A luciferase reporter assay verified that miR-1278 directly targets YAP1 at the predicted site (Figure 5(g)). RIP assays showed that circPTK2, miR-1278, and YAP1 were significantly enriched in the Ago2-precipitated RNA-induced silencing complexes, indicating the presence of the ceRNA axis circPTK2/miR-1278/YAP1 in LSCC cells (Figure 5(h)). RT-qPCR and western blot analyses indicated that knockdown of circPTK2 reduced the mRNA and protein levels of YAP1 in SNU46 and SNU899 cells (Figures 5(i) and 5(j)). Moreover, we identified that circPTK2 overexpression counteracted the suppressive effects of miR-1278 overexpression on YAP1 expression (Figure 5(k)). In summary, circPTK2 acts as a ceRNA for miR-1278 to upregulate YAP1 expression in LSCC cells.

### 3.6. YAP1 Knockdown Inhibits Malignant Phenotypes of LSCC Cells

**Figure 6:**
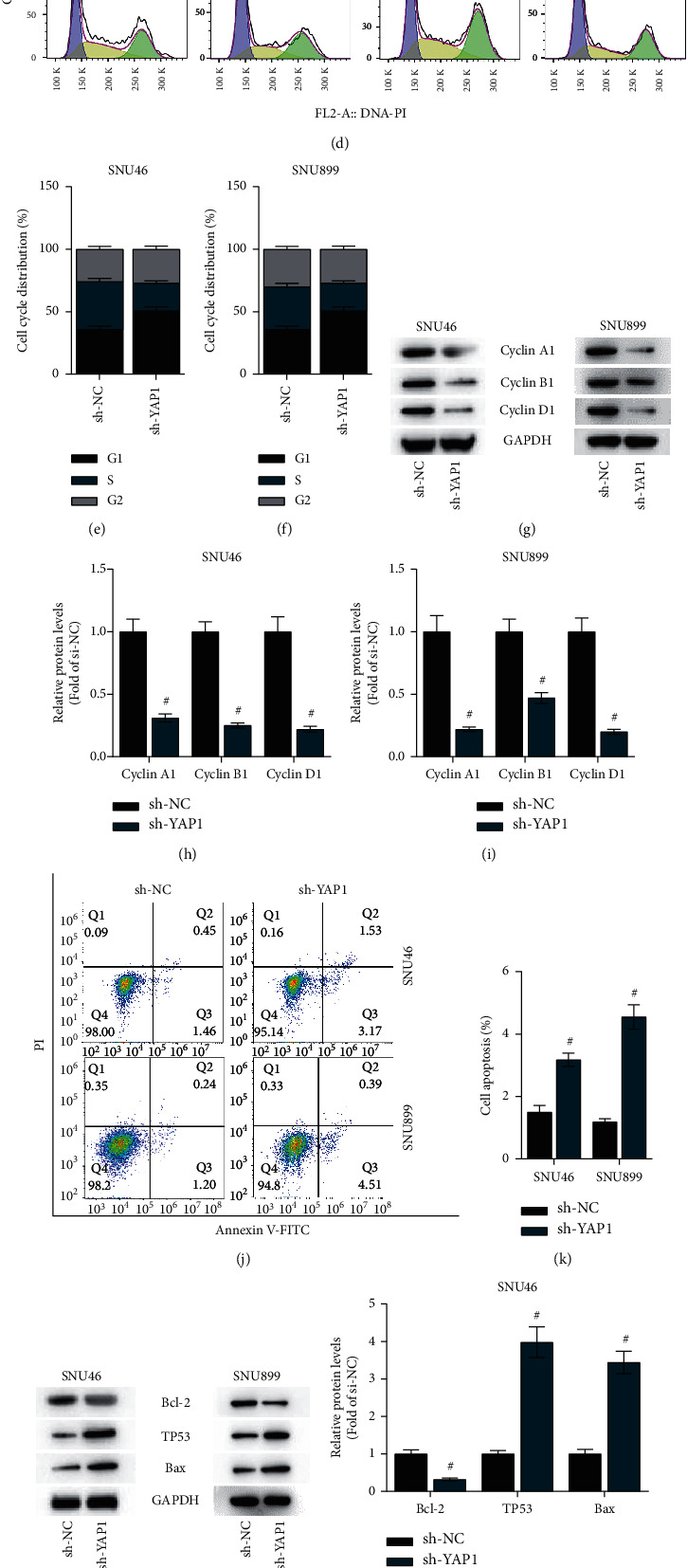
YAP1 knockdown inhibits malignant phenotypes of LSCC cells. (a) RT-qPCR analysis was utilized to examine the knockdown efficiency of circPTK2 in SNU46 and SNU899 cells. (b, c) A CCK-8 assay was conducted to assess the viabilities of SNU46 and SNU899 cells. (d–f) Flow cytometry analysis was carried out to measure the cell cycle in LSCC cells. (g–i) The protein expression of Cyclin A1, Cyclin B1, and Cyclin D1 was measured by western blot analysis. (j, k) The apoptosis of SNU46 and SNU899 cells was evaluated using flow cytometry analysis. (l–n) The protein levels of Bax, TP53, and Bcl-2 were tested by western blot analysis. ^#^*p* < 0.05.

The function of YAP1 in SNU46 and SNU899 cells was examined. We knocked down YAP1 expression using sh-YAP1, and the knockdown efficiency was confirmed by RT-qPCR (Figure 6(a)). As the CCK-8 assay showed, silencing YAP1 suppressed the viabilities of SNU46 and SNU899 cells (Figures 6(b)–6(c)). The cell cycle was also suppressed by YAP1 depletion in LSCC cells (Figures 6(d)–6(f)). Additionally, the levels of cell cycle-associated proteins (Cyclin A1, Cyclin B1, and Cyclin D1) in SNU46 and SNU899 cells were reduced after YAP1 knockdown (Figures 6(g)–6(i)). We then investigated the effects of YAP1 on LSCC cell apoptosis. Flow cytometry analysis showed that silencing YAP1 increases the apoptosis rate of SNU46 and SNU899 cells (Figures 6(j)–6(k)). Furthermore, silencing YAP1 elevated the protein expression of Bax and TP53 but reduced that of Bcl-2 in SNU46 and SNU899 cells (Figures 6(l)–6(n)). Therefore, YAP1 may play an oncogenic role in LSCC.

### 3.7. CircPTK2 Promotes LSCC Progression by Upregulating YAP1

**Figure 7:**
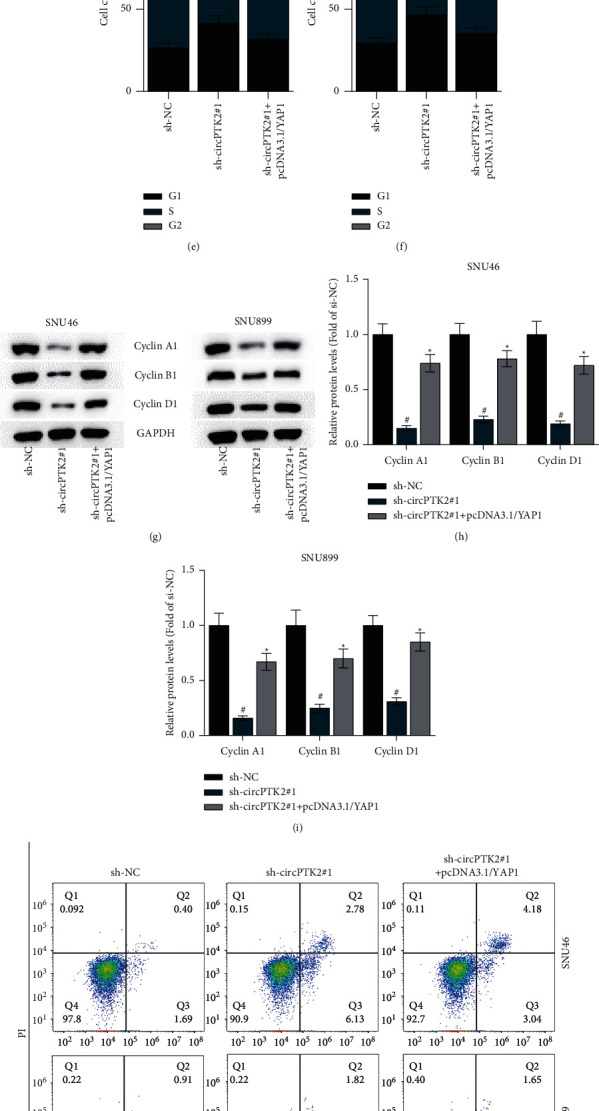
CircPTK2 promotes LSCC progression by upregulating YAP1. (a, b) RT-qPCR and western blot analyses were used to examine the overexpression efficiency of YAP1 in SNU46 and SNU899 cells. (c, d) CCK-8 assay was conducted to assess the proliferation of SNU46 and SNU899 cells. (e, f) Flow cytometry analysis was carried out to measure the cell cycle in LSCC cells. (g–i) Western blot analysis was used to measure the levels of cell cycle markers. (j, k) The apoptosis of SNU46 and SNU899 cells was evaluated using flow cytometry analysis. (l–n) The protein levels of Bax, TP53, and Bcl-2 were tested by western blot analysis. ^#^*p* < 0.05 versus the sh-NC group; ^#^*p* < 0.05 versus the sh-circPTK2#1 group.

We next explored how circPTK2 mediates LSCC progression. RT-qPCR and western blot analyses demonstrated the successful overexpression efficiency of pcDNA3.1/YAP1 in SNU46 and SNU899 cells (Figures 7(a)–7(b)). CCK-8 assay indicated that upregulated YAP1 restored the inhibited cell proliferation by downregulated circPTK2 (Figures 7(c)–7(d)). The suppressive effects of circPTK2 knockdown on the cell cycle were also reversed by YAP1 overexpression (Figures 7(e)–7(f)). Furthermore, YAP1 overexpression restored the levels of cell cycle-related proteins reduced by circPTK2 knockdown (Figures 7(g)–7(i)). Additionally, flow cytometry analysis showed that silencing circPTK2 promoted cell apoptosis, while this effect was weakened after overexpression of YAP1 (Figures 7(j)–7(k)). Likewise, overexpressed YAP1 abated the effects mediated by silenced circPTK2 on cell apoptosis-related proteins (Figures 7(l)–7(n)). Overall, circPTK2 promotes LSCC progression by upregulating YAP1.

## 4. Discussion

CircRNAs have highly conserved sequences with high stability that are widely expressed in mammalian cells and tissues; these characteristics render circRNAs better biomarkers than linear RNAs for diagnosing various diseases [27]. Increasing reports have indicated the significant roles of circRNAs in LSCC progression [28, 29]. This study investigated the functional and regulatory roles of circPTK2 hsa_circ_0003221 in LSCC. Our findings demonstrated that circPTK2 depletion inhibited cell viability and induced cell apoptosis and cell cycle arrest in LSCC. Moreover, inhibition of circPTK2 suppressed xenograft tumor growth *in vivo*. These data indicated that circPTK2 knockdown inhibits LSCC progression. Previous research showed that circPTK2 (hsa_circ_0005273) exerts oncogenic functions in colorectal cancer [20], while circPTK2 (hsa_circ_0003221) facilitates bladder cancer cell proliferation and migration [22]. CircPTK2 (hsa_circ_0008305) is downregulated in non-small cell lung cancer and inhibits epithelial-mesenchymal transition [21]. These three circRNAs (hsa_circ_0005273, hsa_circ_0008305, and hsa_circ_0003221) are derived from the same pre-mRNA PTK2 but have different sequences. hsa_circ_0003221 has a spliced sequence length of 625 nt with five back-spliced exons (exons 3, 4, 5, 6, and 7), and hsa_circ_0008305 has a spliced sequence length of 584 nt with seven back-spliced exons (exons 8, 9, 10, 11, 12, 13, and 14) of PTK2. hsa_circ_0005273 has a spliced sequence length of 357 nt and contains three back-spliced exons (exons 27, 28, and 29). Their distinctive sequences of these circRNAs may lead to their functional differences.

MicroRNAs (miRNAs) are another group of noncoding RNAs that are 19–25 nucleotides in length [30]. The roles of miRNAs in human diseases are indispensable [31]. Increasing evidence has shown that miRNAs act as tumor suppressors in LSCC occurrence and development. For instance, miR-370 interacts with FoxM1 to inhibit LSCC progression [32]. MiR-613 inhibits cell proliferation in LSCC by targeting PDK1 [33]. MiR-143-3p impedes the proliferation, migration, and invasion of LSCC cells by regulating MAGE-A9 [34]. Furthermore, circRNAs can bind to miRNAs to regulate their target mRNAs by competitive interaction [35]. Emerging evidence has suggested that the ceRNA pattern also works in LSCC progression. For example, circRNA_103862 promotes LSCC cell proliferation, migration, and invasion by the miR-493-5p/GOLM1 axis [36], while hsa_circ_0042666 promotes LSCC cell proliferation and invasion by the miR-223/TGFBR3 axis [37]. To further determine the biological functions of circPTK2, we analyzed its ceRNA mechanism in LSCC. We identified the interaction between circPTK2 and miR-1278, as miR-1278 was previously verified to suppress the proliferation and invasion of LSCC cells [38]. The potential antitumor role of miR-1278 has been widely reported. For example, miR-1278 was found to be significantly downregulated in colorectal cancer tissues and cell lines [39], and miR-1278 overexpression indicates a favorable prognosis in osteosarcoma patients [40]. MiR-1278 targets MSI1 to inhibit gastric cancer development [41]. Here, miR-1278 was shown to be downregulated in LSCC cells. Additionally, circPTK2 knockdown upregulated miR-1278 expression, suggesting that circPTK2 may exert ceRNA function by sponging miR-1278 in LSCC cells.

Next, we identified that YAP1 was a downstream target of miR-1278. Numerous studies have shown that overexpression of the YAP1 gene is found in various human cancers. YAP1 can induce epithelial-mesenchymal transition, increase the number of cancer stem cells, and inhibit cell apoptosis *in vitro*, and the abilities of cancer cell invasion, migration, and tumorigenicity in nude mice can be reduced by YAP1 knockdown [42–44]. Moreover, it was reported that the knockdown of YAP1 inhibits cell viability and glycolysis but induces apoptosis in LSCC [45]. Consistent with the previous findings, our study demonstrated that deficiency of YAP1 inhibited cell viability and induced cell apoptosis and cell cycle arrest in LSCC. The rescue experiments showed that YAP1 overexpression reversed the effects of circPTK2 on LSCC cells. Therefore, YAP1 may be an oncogenic gene in LSCC.

In summary, we identified that circPTK2 was upregulated in LSCC cells and promoted malignant phenotypes of LSCC cells by upregulating YAP1. More importantly, circPTK2 downregulation suppressed tumor growth *in vivo*. The present study may provide new insight into therapeutic strategies for LSCC and suggests that circPTK2 may be a potential diagnostic biomarker for LSCC.

## Figures and Tables

**Table 1:** Correlation between hsa_circ_0003221 expression and clinicopathological parameters in LSCC patients.

Characteristics	hsa_circ_0003221 expression		*p* value
	High *n* = 21	Low *n* = 20	
Gende*r*			
Male (27)	16	11	0.153
Female (14)	5	9	
Age			
<60 (18)	10	8	0.623
≥60 (23)	11	12	
Smoking status			
Nonsmoker (17)	9	8	0.853
Smoker (24)	12	12	
Clinical stage			
I/II (23)	8	15	0.017
III/IV (18)	13	5	
T stage			
T1/2 (19)	8	11	0.278
T3/4 (22)	13	9	
Lymph-node metastasis			
Positive (21)	14	7	0.043
Negative (20)	7	13	

^*∗*^ *p* < 0.05 is considered significant (Fisher's exact test).

## Data Availability

The datasets used during the current study are available from the corresponding author on reasonable request.
